# Prevalence and incidence of multiple sclerosis in central Poland, 2010–2014

**DOI:** 10.1186/s12883-016-0662-8

**Published:** 2016-08-11

**Authors:** Waldemar Brola, Piotr Sobolewski, Stanisław Flaga, Małgorzata Fudala, Wiktor Szczuchniak, Jan Stoiński, Anita Rosołowska, Jacek Wójcik, Katarzyna Kapica-Topczewska, Danuta Ryglewicz

**Affiliations:** 1 Department of Neurology, Specialist Hospital in Końskie, 41 Gimnazjalna Street, 26-200 Końskie, Poland; 2 Department of Neurology, Holy Spirit Specialist Hospital in Sandomierz, 13 Schinzla Street, 27-600 Sandomierz, Poland; 3 AGH University of Science and Technology, 30 Mickiewicza Av., 30-059 Krakow, Poland; 4 Department of Neurology, Regional Hospital, 1 Szpitalna Street, 26-110 Skarżysko-Kamienna, Poland; 5 Department of Neurology, Regional Hospital, 70 Radomska Street, 27-200 Starachowice, Poland; 6 Department of Neurology, Regional Hospital, 78 11-Listopada Street, 28-200 Staszów, Poland; 7 Department of Neurology, Medical University of Bialystok, 24A Skłodowskiej – Curie Street, 15-276 Bialystok, Poland; 8 First Department of Neurology, Institute of Psychiatry and Neurology, 9 Sobieskiego Street, 02-957 Warsaw, Poland

**Keywords:** Multiple sclerosis, Prevalence, Incidence, Epidemiology, Poland

## Abstract

**Background:**

Comprehensive epidemiologic data for multiple sclerosis (MS) in Poland are limited. The aim of this cross-sectional population-based study was to determine the incidence and prevalence of MS in the Swietokrzyskie Region (central Poland).

**Methods:**

This study identified MS cases every year between 1 January 2010 and 31 December 2014. The study area population on the prevalence day (December 31, 2014) was 1,263,176 (646,506 women and 616,670 men). A total of 1462 patients with a clinically definite diagnosis of MS according to McDonald’s criteria (2005), recorded in the Polish Multiple Sclerosis Registry, were considered for estimation of crude, age- and sex-specific prevalence, and incidence.

**Results:**

The overall crude prevalence rate of confirmed MS patients was 115.7/100,000 (95 % confidence interval (CI), 111.2–121.4). A significantly higher prevalence was recorded in females (159.6/100,000; 95 % CI, 151.1–165.3) than in males (69.7/100,000; 95 % CI, 62.4–77.3) (*P* < 0.001). Age-adjusted rates for the Polish and European Standard Population were 109.8/100,000 (95 % CI, 105.4–114.8) and 106.6/100,000 (95 % CI, 101.1–111.2), respectively. The female/male ratio was 2.4. The mean annual incidence was 4.2/100,000 (95 % CI. 3.7–4.4).

**Conclusion:**

The incidence and prevalence of MS in the Swietokrzyskie region confirm that central Poland is a high risk area for MS. Compared with previous epidemiologic studies from Poland, the prevalence of MS has increased during recent years.

## Background

Multiple sclerosis (MS) is one of the most common causes of neurological disability in young people [1]. The number of MS patients worldwide exceeds 2.3 million, of whom approximately 600,000 live in Europe [2]. It is estimated that there are 40,000–50,000 MS patients in Poland, and the prevalence is estimated to be between 37 and 91 cases per 100,000 citizens [3]. Most of the epidemiological studies of MS in Poland were conducted many years ago, and provide data from only some regions of Poland [4–13]. In addition, the results of most of the studies were published only in local Polish language scientific literature and are not available to the rest of the world. In the annually published atlas of MS, Poland has been an uncharted territory for many years [2]. For a long time, no registry of patients with MS existed in Poland.

Systematic collection of epidemiological data on MS was initiated in Poland in 2010. With the goal of long-term observation of patients with MS, a team of information technology specialists from the AGH University of Science and Technology in Krakow designed a computer program that became the basis for the Polish MS Registry (RejSM). All Departments and Wards of neurology, rehabilitation centers, clinics and private neurological offices were invited to participate. The registration of patients began in the Swietokrzyskie Voivodeship (central Poland). The data categories that were collected included patient age, sex, family status, place of residence, education, family history, and information directly related to the disease itself, such as the date of onset, type of first symptoms, date of diagnosis, type of disease, comorbidities, occurrence of relapses, additional examinations and tests (magnetic resonance imaging (MRI), cerebrospinal fluid test, and evoked potentials), Expanded Disability Status Scale (EDSS) results, and types of treatment (modifying the course of the disease and symptomatic treatment). Since 2013, the RejSM has been an all-Poland project. However, the only region where full data have been collected is the Swietokrzyskie Voivodeship. This study investigated the prevalence, incidence, and other epidemiological data of MS in this region of central Poland. The data were also compared with the results obtained earlier for other regions of Poland.

## Methods

### Area of investigation

**Fig. 1:**
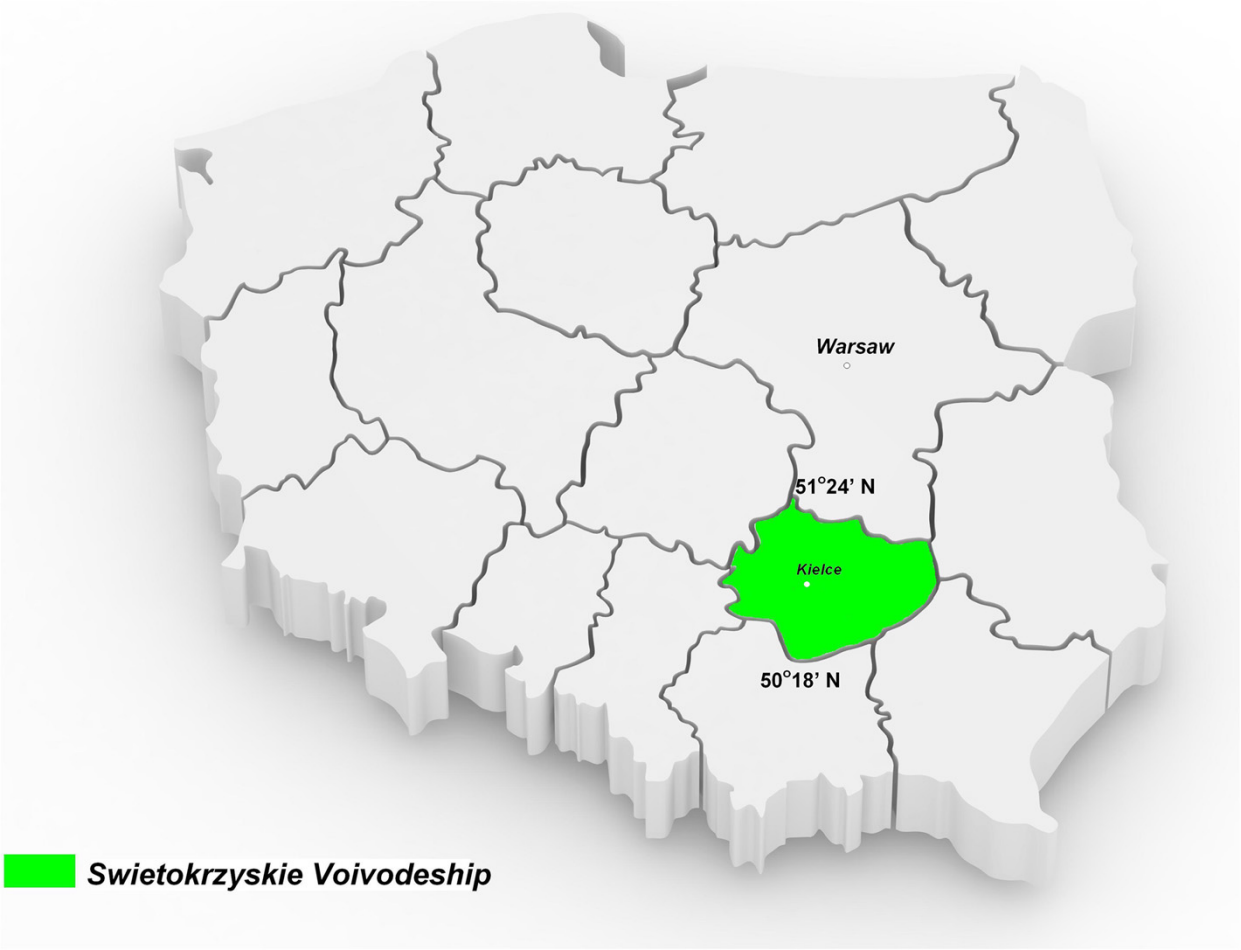
Map of Poland showing the location of the Swietokrzyskie Voivodeship. Latitude between 51.24 and 50.18 N

The survey was conducted in the Swietokrzyskie Voivodeship (located in the center of Poland) between 51°34’ and 50°18’ north latitude and 19°70’ and 21°87’ east longitude (Fig. 1). It covers an area of 11,710.50 km^2^ and comprises 31 towns and 2542 villages at a mean altitude of 243 m above sea level; the climate is temperate. The population on the selected prevalence day (December 31, 2014) was 1,263,176 inhabitants (616,232 men and 646,506 women) [14]. The region is ethnically and culturally homogenous.

This study was performed at the MS Center of the Department of Neurology, Specialist Hospital in Końskie, and seven main hospital centers in the Swietokrzyskie region.

### Ethical approval

Informed consent was obtained from each participant or the next of kin before any interview or neurological examination was conducted. All individual data were automatically anonymized by replacement of the personal identity numbers (PESEL - Powszechny Elektroniczny System Ewidencji Ludności; Universal Electronic System for Registration of the Population) with unique number codes for use in the study. The study was approved by the Regional Medical Ethics Committee of the Swietokrzyskie Medical Council in Kielce.

### Study design

The web-based version of the RejSM (http://www.rejsm.pl) was started in 2010. Patients with MS, according to the 2005 McDonald criteria [15], were prospectively and retrospectively registered and followed at each visit. PESEL numbers (11-digit unique identity numbers mandatory in Poland since 1979), were relevant to the study because they ensured that MS patients registered by different researchers were counted only once in the survey. The data were recorded by an experienced neurologist of the RejSM. All participating centers took responsibility for their data and performed source data monitoring. The MS specialists responsible for data collection had been previously trained in data collection, patient monitoring, and treatment procedures. The data from the RejSM were validated by verifying data concordance between the electronic database and medical documentation. In the case of incomplete or unclear information, the coordinators contacted the data providers by telephone and interviewed them to confirm the details. Next, within 1 year of the prevalence day, all subjects were clinically examined by a neurologist specializing in MS to verify the diagnosis of MS according to McDonald’s criteria. The patients were evaluated with the EDSS proposed by Kurtzke [16]. All patients with a definite diagnosis of MS who were born and lived in the Swietokrzyskie Voivodeship were recruited into the study on the prevalence day (December 31, 2014). A total of 1462 MS patients were included in the estimation of prevalence and incidence.

### Statistical analysis

Prevalence was based on the number of MS patients registered in the RejSM who were residents of the Swietokrzyskie region on the prevalence date of December 31, 2014.

Prevalence was expressed as the number of all cases on the prevalence day divided by the population of the Swietokrzyskie Voivodeship on the same day (available in the Demographic Yearbook of Poland 2015) [14]. Crude sex and age area-specific prevalences were calculated as the number of cases on prevalence day per 100,000 inhabitants. The prevalence of MS was adjusted by a direct method, using the Polish and European population as a standard [17]. The incidence was calculated annually (on 31^st^ December) using the number of newly diagnosed cases each year from 1^st^ January to 31^st^ December as the numerator and the size of the population as per the official data every year from 2010 to 2014, according to the Polish Central Statistical Office [14]. The mean incidence was then calculated as a mean of the individual yearly values in the 5-year period.

The 95 % confidence intervals (CIs) for prevalence and incidence were calculated using the Poisson distribution. Significant differences between groups were tested by unpaired *t*-tests and the Chi-square test or Fisher exact test, as appropriate. *P*-values <0.05 were considered statistically significant. The statistical analysis was performed using STATISTICA software, version 8.0 (2007; StatSoft, Inc.).

## Results

A diagnosis of MS was confirmed by a neurologist or MS specialist over the 5-year study period. On the prevalence day (December 31, 2014), there were 1462 MS patients (430 men and 1032 women; mean age: 43.3 ± 11.9 years) living in the study area. The female-to-male ratio was 2.4:1. Demographic and clinical characteristics of these patients on the prevalence day are shown in Table 1. The mean duration of MS from onset to the prevalence day was 15.5 ± 9.8 years (range: 0–52 years). The mean length of time between onset of the first symptoms and diagnosis was 28.8 ± 56.2 months, with a median of 22 months (range, 1–168 months). According to Kurtzke’s EDSS, 61.5 % of the patients enrolled for the prevalence estimate had an EDSS score between 0 and 3.0, 17.2 % were between 3.5 and 5.0, and 21.3 % were between 5.5 and 8.5. The mean EDSS score was 3.4 ± 2.18 (range: 0–8.5) (mean EDSS for females: 3.6 ± 2.2; for males: 3.2 ± 2.3).

**Table 1:** Clinical and socio-demographic characteristics of the study patients^a^

Variable	Patients with MS
	(*n = 1462*)
Male, *n* (%)	430 (29.4)
Female, *n* (%)	1032 (70.6)
Age, years, mean ± SD (range)	
At the prevalence day	43.3 ± 11.9 (18–78)
At disease onset	29.8 ± 8.6 (16–56)
Initial signs at onset, *n* (%)	
Pyramidal	365 (25.1)
Sensory	161 (10.8)
Visual	251 (17.2)
Brainstem	115 (7.9)
Cerebellar	74 (5.1)
Spinal cord	65 (4.4)
Polysymptomatic	432 (29.5)
Disease course subtypes, *n* (%)	
Relapsing remitting	997 (68.2)
Secondary progressive	317 (21.7)
Primary progressive	148 (10.1)
Treatment during the follow-up period, *n* (%)^b^	341 (23.3)
Interferon beta	227 (15.5)
Glatiramer acetate	79 (5.4)
Selective immunosuppressants (natalizumab and fingolimod)	35 (2.4)
Educational level, *n* (%)	
Higher	212 (14.5)
Secondary	763 (52.2)
Elementary	487 (33.3)

^a^Summary statistics are mean ± standard deviation

^b^Treatment data were collected at the final study visit (31 December 2014)

*MS* multiple sclerosis

The overall crude prevalence in the Swietokrzyskie Voivodeship population of 1,263,176 was 115.7/100,000 (95 % CI, 111.2–121.4). A significantly higher prevalence was recorded in females (159.6; 95 % CI, 151.1–165.3) than in males (69.7; 95 % CI, 62.4–77.3; *P* < 0.001). The age-adjusted prevalence standardized to the Polish population was 109.8 per 100,000 (95 % CI, 105.4–114.8), and the age-adjusted prevalence for the European Standard Population was 106.6 per 100,000 (95 % CI, 101.1–111.2). Table 2 shows the age- and sex-specific prevalence per 100,000 population on December 31, 2014. The age-specific prevalence peaked in the age group 35–44 years. The prevalence was found to increase in the 25–34 year and 35–44 year age groups in males and the 35–44 year and 45–54 year age groups in females, after which it decreased. Significantly more female patients than male patients were diagnosed with definite MS during the study period (*P* < 0.001).

**Table 2:** Age- and sex-specific prevalence of MS per 100,000 inhabitants in the Swietokrzyskie Voivodeship on 31 December 2014^a^

Age (years)	Male				Female				Total			
	Cases	Population	Prevalence	95 % CI	Cases	Population	Prevalence	95 % CI	Cases	Population	Prevalence	95 % CI
0–14	0	90,120	-	-	0	85,469	-	-	0	175,589	-	-
15–24	20	79,053	25.3	19.2–33.4	46	75,319	61.1	54.4–69.8	66	154,372	42.8	36.2–48.9
25–34	117	100,886	115.9	102.3–128.3	259	93,488	277.1	262.4–291.5	376	194,374	193.4	178.6–209.4
35–44	141	92,664	152.2	144.6–159.4	298	87,008	342.5	326.8–358.6	439	179,672	244.3	232.2–258.6
45–54	85	79,912	106.4	96.8–113,2	266	78,822	337.4	322.6–352.2	351	158,734	221.1	212.2–232.5
55–64	50	92,527	54.1	48.6–61,2	138	98,200	140.5	132.6–150.2	188	190,727	98.6	92.4–106.5
≥65	17	81,503	20.8	16.4–26.5	25	128,205	19.5	14.6–25.8	42	209,708	20.0	15.9–24.8
Total	430	616,670	69.7	62.4–77.3	1032	646,506	159.6	151.1–165.3	1462	1,263,176	115.7	111.2–121.4

*CI* confidence interval

Age-adjusted prevalence for the European Standard Population was 106.6/100,000 (95 % CI, 101.1–111.2)

^a^Based on data provided by Polish Central Statistical Office

The incidence of MS was estimated for the period between January 1, 2010 and December 31, 2014, and was calculated annually (on 31^st^ December) (Table 3). During that period, 267 patients (190 women and 77 men) had onset of MS while living in the study area. The clinical course of the newly diagnosed patients consisted of 249 relapsing-remitting and 18 primary progressive patients. The mean age at onset was 30.8 ± 9.6 years (32.1 ± 8.9 years for women and 28.5 ± 8.8 years for men). The crude annual incidence of MS in this population was 4.2 (95 % CI, 3.7–4.4) per 100,000 per year over the 5-year period (5.9 for women and 2.5 for men), and age-standardized rates (adjustment to the European population) of 4.12 [3.32–5.14], 5.68 [4.45–7.82], and 2.21 [1.12–3.31], respectively. The incidence rate for women was 2.4 times higher than for men. The highest rates for both sexes were noted in the group between 25 and 34 years of age. The incidence of MS increased in women from 3.5 in the first year of the study (2010) to 8.2 in the last year of observation (2014). In contrast, there was no significant increase in men (from 2.3 to 3.1).

**Table 3:** Incidence of MS per 100,000 population in the Swietokrzyskie Voivodeship patients between 2010 and 2014^a^

Years	Total				Male				Female			
	Cases	Population	Incidence	95 % CI	Cases	Population	Incidence	95 % CI	Cases	Population	Incidence	95 % CI
2010	37	1,266,014	2.92	2.42–3.55	14	616,462	2.27	1.21–3.04	23	649,552	3.54	2.64–5.14
2011	49	1,278,116	3,83	2.94–5.64	13	624,269	2.08	1.42–3.22	36	653,847	5.51	4.12–7.21
2012	51	1,273,995	4.00	2.95–5.72	13	622,370	2.09	1.38–3.18	38	651,625	5.83	4.46–7.34
2013	58	1,268,239	4.57	3.86–6.12	18	619,232	2.91	1.95–3.84	40	649,007	6.16	5.32–7.89
2014	72	1,263,176	5.70	4.83–6.94	19	616,670	3.08	2.35–4.12	53	645,506	8.21	7.14–8.94

The mean annual incidence, based on a 5-year period of observation, was 4.20 (95 % CI, 3.69–4.42) per 100,000 inhabitants, 2.46/100,000 among men and 5.85/100,000 among women. The incidence rate for women was 2.38 times higher than for men

Age-adjusted incidence rates for European population were 4.12 (3.32–5.14), 5.68 for women (4.45–7.82), and 2.21 for men (1.12–3.31), respectively

^a^Based on data provided by Polish Central Statistical Office

## Discussion

Poland belongs to a group of countries in a geographical area that has a high prevalence of MS. Pugliatti reported that the mean prevalence for Europe during the last 30 years was 83/100,000 [18]. In Poland, there have been no epidemiological studies of MS that included the entire country, and the MS prevalence reported in various regional studies ranged from 37 to 91 per 100,000 residents [3]. It should be noted that most of these studies were conducted many years ago, and their results were most often published in Polish literature only. Epidemiological rates were specified for selected areas and cities of central-western, north-western, and eastern Poland. To date, no observations have been conducted in central Poland. In our study, the prevalence on the prevalence day was 115.7/100,000 (159.6/100,000 for women and 69.7/100,000 for men), which is higher than that presented in previous Polish studies. The first epidemiological studies conducted by Cendrowski in the 1950s in Bydgoszcz and Krosno showed a relatively low prevalence of MS (43 and 37/100,000) [4]. A comparison of MS prevalence in Great Poland (western part of the country) in 1965 and 1981 showed a decrease from 65 to 45 per 100,000 citizens [7]. Later studies conducted in selected regions of Poland indicated that the prevalence of MS may be higher, between 55 and 75 patients per 100,000 residents [9–12]. A number of reports published during the last several years suggest that the prevalence may be even higher. For example, studies conducted in selected cities and regions (Szczecin, Gniezno) have revealed that the MS rate was as high as 90–110/100,000 [10, 19]. In some regions, recognized as higher risk areas for MS, the prevalence was 110–130/100,000 [13, 20, 21]. Characteristic fluctuations in MS prevalence in the regions over time have occurred; for example, the MS prevalence in Gniezno has been recorded as 53.4 (1965), 122.8 (1982), 87.9 (1992), and 97.8/100,000 (1999) [20]. However, it is difficult to conclude whether the fluctuations pertain only to some regions or if this is a more general epidemiological tendency, particularly because the study included only approximately 10 % of the national area and population of the country.

The geographical location of Poland suggests that the prevalence of MS should be similar to that in neighboring countries. In Germany, the prevalence ranges between 70 and 95/100,000, but significant differences among various regions are observed [22–28]. An analysis in Germany in the early 1980s showed variations in prevalence: Halle (43/100,000) [23], Rostock region (69/100,000) [24], and Göttingen (83/100,000) [25]. In the 1990s, an increase in prevalence was observed, at 85/100,000 in south Hesse [26], 95/100,000 in the city of Bochum [27], and the highest rate was observed in the region of South Saxony (108/100,000) [28]. A similar analysis in the Czech Republic showed a prevalence of 70.8/100,000 and significantly higher rates for the regions of Teplice and Chomutov (160.0 and 103.0/100,000, respectively) [29]. The prevalence in Estonia in 1989 was estimated to be 51/100,000 and was the highest among patients aged 35–49 years [30]. In Latvia, in the 1960s, the prevalence ranged between 38 and 85/100,000 [31]; in Lithuania, in the 1980s, it was 35/100,000, and in Belarus it ranged between 20 and 55/100,000 [31]. In central and south-western Ukraine, the prevalence in 2001 was estimated to be 41/100,000 [31].

Alonso and Hernán analyzed worldwide epidemiological data from the period between 1966 and 2007, and estimated that the MS incidence rate was 3.6/100,000 in women and 2.0/100 000 in men [32]. They concluded that the highest incidence rates were observed in Seinajoki (Finland); in the period between 1979 and 1993, the rates were 10.3 and 6.2 /100,000 for female and male residents, respectively. The lowest incidence was recorded in Queensland (Australia), where in the period between 1971 and 1981, the rate was only 1.5/100,000 for women and 0.6/100,000 for men [32]. In Europe, the rate in the overall population ranged between 3.5 and 5.5/100,000 (mean 4.3/100,000) [33].

The annual MS incidence rate in Poland is estimated to be 2.4–4.3/100,000 residents, which equates to approximately 1300–2100 new MS cases nationally each year [3]. In our study, the mean incidence rate was 4.2 per 100,000/year. Between 2010 and 2014, a gradual increase in incidence among women (3.5/100,000/year in the first year of the study [2010] to 8.2 in the last year of observation [2014]) was observed. Such a trend was not observed in men (no significant increase from 2.3 to 3.1). However, the time of observation was too short for a thorough analysis of the trend in incidence, and the reasons behind any increase. Our registry started functioning in 2010, and an increase in incidence rates could be related to constantly improving methods of gathering and analysis of data, as well as greater involvement of the facilities included in our research. We anticipate that RejSM will continue to develop through the coming years, which will allow long-term observation of MS patients and more detailed analysis, as well as more precise estimation of prevalence and incidence.

The mean incidence rate in our study was higher than that in most previously reported studies. Studies of MS incidence in Poland were initiated by Cendrowski, who reported an incidence of 1.2/100,000 in Pruszków in the years 1937–1960 [4]. Later studies conducted in other regions of Poland reported higher rates. In the period between 1960 and 1992, the incidence rate in the region of Szczecin was 3.7/100,000/year, while in the period between 2000 and 2005, the rate decreased to 2.44/100,000/year [9]. In Great Poland, in the years between 1979 and 1981, this rate was found to be 3.7/100,000 [7]. Since the 1960s, systematic epidemiological studies have been conducted in Gniezno. It was found that in the period between 1965 and 1999, the mean incidence rate of MS in this area was 3.7/100,000/year [20].

Similar incidence rates have been observed in neighboring countries. In Germany, the MS incidence rate in the period between 1979 and 1992 was estimated to be 4.2/100,000 [18, 22]. The highest incidences of MS (8.0/100,000/year) were observed in the urban region of Erfurt (Thuringia) in the period between 1998 and 2002 [34], and in Bochum (6.1/100,000/year) [27]. In the Czech Republic, the incidence of MS was estimated to be between 4 and 8/100,000/year in the period between 1985 and 1990 [29], while in the Ukraine the rate was 0.7/100,000/year (1990–1994) [35].

In our study, female MS cases were dominant (female-to-male ratio 2.4). In Szczecin, this rate was 1.46 [3]. Similar results were obtained in a study in Lublin (female-to-male ratio 2.24) [12], a Polish multicenter study carried out in 2009 (2.4) [36], and in nearby countries such as Denmark, Sweden, and Germany (2.2, 2.35, and 2.5/100,000, respectively) [22].

There may be several factors contributing to the higher prevalence and incidence rates of MS in this study than in previously published studies in Poland. First, all of the previous studies were conducted using the door-to-door method, which is laborious, time-consuming, and makes collection of an entire dataset rather unrealistic. Collection of data performed by experienced neurologists from specific regions, information archived in an electronic MS registry, systematically conducted follow-ups, and uniform data processing ensure a significantly better insight into the current epidemiological situation. Introduction of routine MRI examinations, evoked potentials, or oligoclonal band tests have also improved the diagnostic evaluation. These advances have resulted in, among other things, a significant shortening of the time between the first symptoms and diagnosis. More precise criteria for the diagnosis of MS (McDonald’s 2005 and 2010), allowing many cases of suspected MS to be qualified as certain, also contributes to the efficacy of MS treatment.

This study has a number of limitations. The oldest and most disabled patients may not have been identified. The number of elderly patients with long-term disease, especially secondary progressive MS, may be underestimated. Difficulties in reaching people who rarely have any contact with medical services or who attend their GP rather than a neurologist may result in omission from the registry, which is an issue for all epidemiological registries. Long-term observation and an aim for complete identification of all patients is desirable for accurate estimation of prevalence and incidence. Additionally, some young patients with mild forms of MS could have avoided or not required contact with the health service during the years of this study. However, systematic implementation of the registry during the next several years should allow us to identify the entire population of MS patients, not only from the Swietokrzyskie Voivodeship, but also from other regions, or ideally all of Poland. Our RejSM system is continuously growing, and a collaboration with the National Health Fund should soon produce results as well as ensure that Poland is no longer an uncharted territory on the MS epidemiological map of Europe.

## Conclusion

The results obtained are the outcome of the first national Polish MS patient registry. The levels of incidence and prevalence in the Swietokrzyskie region confirm that central Poland is a high risk area for MS. In comparison with previous epidemiologic studies from Poland, the incidence and prevalence of MS has risen in the recent years. To fully determine the epidemiology of MS in Poland, further systematic long-term research programs including other regions of the country are needed.

## Abbreviations

CI, confidence interval; EDSS, Expanded Disability Status Scale; MRI, magnetic resonance imaging; MS, multiple sclerosis; PESEL, Powszechny Elektroniczny System Ewidencji Ludności, Universal Electronic System for Registration of the Population; RejSM, Polish MS Registry
